# ﻿Evolution of connective glands reveals a new synapomorphy for Malpighiaceae and the hidden potential of staminal glands for Malpighiales systematics

**DOI:** 10.3897/phytokeys.232.110162

**Published:** 2023-09-15

**Authors:** Rafael Felipe de Almeida, Gustavo Arévalo-Rodrigues, Isa L. de Morais, Poliana Cardoso-Gustavson

**Affiliations:** 1 Universidade Estadual de Goiás, Quirinópolis, Goiás, Brazil Universidade Estadual de Goiás Quirinópolis Brazil; 2 Royal Botanic Gardens, Kew, Richmond, Surrey, UK Royal Botanical Gardens Richmond United Kingdom; 3 Instituto de Pesquisas Ambientais, São Paulo, São Paulo, Brazil Instituto de Pesquisas Ambientais Sao Paulo Brazil; 4 Universidade Federal do ABC, São Bernardo do Campo, São Paulo, Brazil Universidade Federal do ABC São Paulo Brazil

**Keywords:** Anther, character-mapping, flowering plants, Rosids, secretory epidermis

## Abstract

Connective glands are important morphological characters for the taxonomy of some genera of Malpighiaceae, with few recent studies having just elucidated these glands’ anatomical and ecological functions. In order to test the systematic relevance of connective glands to the currently accepted phylogenetic informal clades of Malpighiaceae, we characterised the anatomy and/or histochemistry of two-thirds of Malpighiaceae genera and ten species from nine families of Malpighiales to test: 1. Do connective glands occur in the flowers of all informal clades of Malpighiaceae?; and 2. Are they taxonomically relevant to characterise those clades? We sampled 25 genera and 26 species of Malpighiaceae, processing their anthers using traditional anatomical methods and characterising their glands using light microscopy and SEM imaging. Selected species were subjected to histochemical tests, and an additional 21 genera and 33 species of Malpighiaceae and nine families (ten species) of Malpighiales were included in our sampling from the literature. Three anatomical characters were scored, coded and mapped using Maximum Likelihood methods onto the molecular phylogeny of Malpighiaceae. All sampled species of Malpighiaceae showed connective glands characterised as epidermal or trichomal elaiophores. Our character-mapping analyses recovered connective elaiophores as a new synapomorphy for Malpighiaceae. Different types of epidermal or trichomal elaiophores were recovered as homoplasies for the *Christianella* and *Banisteriopsis* clades and the genera *Byrsonima*, *Camarea* and *Cottsia*. Our analyses also recovered the glands’ place of insertion in the stamen and the exudate type as potential new synapomorphies or homoplasies for the families of Malpighiales sampled. Our results propose the connective elaiophores as a new synapomorphy for Malpighiaceae and hypothesise the role that different staminal glands might play in the systematics of Malpighiales. Further comprehensive anatomical studies are still needed for the staminal glands of most families of this order to shed new light on the patterns recovered in our study.

## ﻿Introduction

Malpighiaceae (Malpighiales) are a family of flowering plants comprising 75 genera and 1,350 species of trees, shrubs, subshrubs and lianas distributed across tropical and subtropical regions of the world (Almeida et al. 2020; POWO 2023). The monophyly of this family has been corroborated by several molecular phylogenetic studies of the past two decades (Cameron et al. 2001; Davis et al. 2001; Davis and Anderson 2010; Almeida and van den Berg 2021; Almeida et al. 2023), but the monophyly of its subfamilies, most tribes, and several genera was not supported (Cameron et al. 2001; Davis et al. 2001; Davis and Anderson 2010; Almeida and van den Berg 2021). Since then, several new genera and generic synonymies have been gradually proposed to accommodate these newly identified lineages (Anderson 2006; Anderson and Davis 2007; Almeida and van den Berg 2021). Although no new classification system based on phylogenetic evidence has ever been proposed for Malpighiaceae, the family is currently divided into ten informally named clades: 1. Byrsonimoids, 2. Acridocarpoids, 3. Mcvaughioids, 4. Barnebyoids, 5. Ptilochaetoids, 6. Bunchosioids, 7. Hiraeoids, 8. Tetrapteroids, 9. Stigmaphylloids, and 10. Malpighioids (Davis and Anderson 2010; Almeida and van den Berg 2021; Almeida et al. 2023).

**Figure 1.**
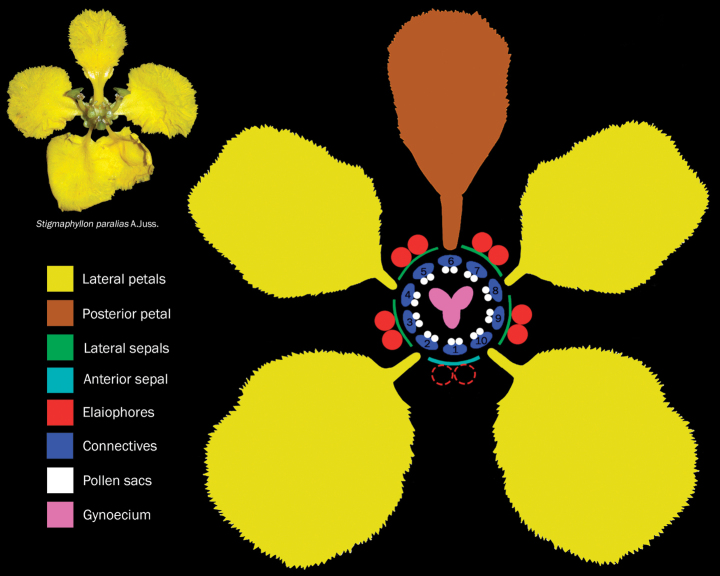
Diagram of a Malpighiaceae flower showing all informative floral organs. Upper left photograph of *Stigmaphyllonparalias* A.Juss. by Marco Pellegrini.

Neotropical Malpighiaceae show a conspicuous floral conservatism characterised by monosymmetric (i.e., zygomorphic), monoecious flowers with five sepals adnate at the base, abaxially (i.e., to the flower axis) bearing a pair of oil-secreting glands (i.e., elaiophores) near the base (sometimes absent from the anterior sepal or completely absent in few genera; Fig. 1). The five petals are free, clawed (i.e., narrowed at base), and divided into groups: four lateral petals and a single posterior petal slightly different from the lateral ones (i.e., in size, colour, shaped, posture, or margin; Fig. 1). The androecium comprises two whorls of (1–)5 stamens with eglandular filaments connate at base, connectives frequently glandular and hairy, with anthers basifixed and composed by two, rimose (porate in *Coleostachys* A.Juss.) pollen sacs (Fig. 1). The gynoecium consisting of a 3-carpellate and 3-locular ovary, the ovary showing primordial projections in the genera bearing ornamented mericarps (which will later fully develop into wings, winglets or setae); style (1–)3 usually free and long, cylindrical to flattened, eglandular and usually glabrous, with the apex truncate, rounded, uncinate or expanded (i.e., with a leaf-like projection); and each style with one, capitate or punctate, terminal or lateral stigma (Fig. 1). The fruits vary greatly, ranging from indehiscent fleshy (i.e., drupes), indehiscent dry (i.e., nuts), to dehiscent dry fruits splitting into three fruitlets (i.e., schizocarps) smooth or ornamented (i.e., winged or setose) mericarps (Anderson 1979; Almeida et al. 2020).

The floral conservatism of Malpighiaceae is the result of a 75-million-year mutualism with certain groups of bees that collect the non-volatile oil produced by their elaiophores (Michener 2007; Davis et al. 2014). The solitary female bees collect oil from elaiophores using their posterior legs for larval provisioning and breeding cell lining of their nests (Simpson and Neff 1981; Buchmann 1987; Vogel 1990; Michener 2007; Reis et al. 2007). Malpighiaceae are the oldest and most diversified flowering plants to offer oil as a floral reward to their pollinators (Renner and Schaefer 2010). Their ancestor emerged ca. 75 million years ago alongside lineages of oil-collecting bees, and both remained on an exclusive mutualism for at least 40 million years when new lineages of oil-offering angiosperms emerged (e.g., Iridaceae, Krameriaceae, and Plantaginaceae; Renner and Schaefer 2010; Davis et al. 2014; Martins and Melo 2016). Neotropical species of Malpighiaceae are pollinated mainly by Centridini, Tapinotaspidini, and Tetrapediini bees (Vogel 1974; Alves-dos-Santos et al. 2007; Martins and Melo 2016). On the other hand, the Paleotropical species of Malpighiaceae show a conspicuous change in their pollination syndrome, from oil-offering to pollen-offering, since Centridini, Tapinotaspidini, and Tetrapediini bees are only found in the New World (Davis et al. 2014). These Old-World lineages mostly show actinomorphic, functionally dioecious flowers without oil-secreting glands or with glands reduced to 1–3 large nectaries similar in shape and anatomical structure to the elaiophores of Neotropical species (Anderson 1979; Davis et al. 2014; Guesdon et al. 2019).

Elaiophores are floral glands consisting of a uniseriate and columnar secretory epithelium with a thick cuticle and a parenchyma that is vascularised by xylem and phloem that produce and secrete non-volatile oils as a reward for their pollinators (Vogel 1974). Oil-secreting glands occur in 1500–2400 species of 11 families of monocots and eudicots, including Malpighiaceae. Most of these families only show a single type of elaiophore: 1. epidermal or 2. trichomal (Vogel 1974; Renner and Schaefer 2010). The monocot families Iridaceae and Orchidaceae are currently the only ones showing both epidermal and trichomal elaiophores (Renner and Schaefer 2010).

Over the past three decades, the epidermal elaiophores of Malpighiaceae have been anatomically and ecologically studied by different authors (Lobreau-Callen 1989; Simpson 1989; Subramanian et al. 1990; Vogel 1990; Cocucci et al. 1996; Carvalho et al. 2005; Araújo and Meira 2016; Possobom and Machado 2017; Torretta et al. 2018; Avalos et al. 2020; Aliscioni et al. 2022). Nonetheless, elaiophores were only recently found in additional Malpighiaceae floral organs besides their sepals. Possobom et al. (2015) identified epidermal glands in the connectives of *Diplopteryspubipetala* (A.Juss.) W.R.Anderson & C.C.Davis and revealed the non-volatile lipidic nature (i.e., elaiophores) of the exudate of these staminal glands. Possobom et al. (2015) also hypothesised that the function of these staminal elaiophores is to increase pollen transfer efficiency and chemically attract pollinators. The occurrence of glandular projections in stamen’s connectives of Malpighiaceae has been noticed by several authors since incredibly early in the taxonomy of several genera of this family (Anderson 1975, 1982, 1987, 1995; Gates 1982; Johnson 1986; Niedenzu 1928; Simpson 1989). Nonetheless, it was only recently that the structure, nature, and occurrence of staminal glands in Malpighiaceae had their systematic relevance properly tested in a phylogenetic context with the study of Arévalo-Rodrigues et al. (2020). These authors described the occurrence of staminal elaiophores for all analysed genera from the Stigmaphylloid clade and the outgroup species *Byrsonimaspicata* (Cav.) DC.

Thus, aiming to better characterise the macro-evolutionary patterns of the connective glands of Malpighiaceae, we performed a broad micromorphological study, including 46 genera from nine of the ten informal clades of the family (*sensu*Almeida and van den Berg 2021). Our main goal was to identify the nature of the connective glands of 46 genera and 59 species of Malpighiaceae, from both the New and Old World, by characterising their anatomy, exudate, and tissue histochemistry. More specifically, we intended to answer the following questions: 1. Do staminal connective glands occur in the flowers of all ten informal clades of Malpighiaceae? 2. Are they taxonomically relevant to characterise those clades?

## ﻿Methods

### ﻿Plant material

Anthers of flowers at anthesis were sampled from herbarium specimens or collected in the field and fixed in FAA 50 (Johansen 1940) for 25 genera and 26 species of Malpighiaceae (Table 1). Herbarium samples were submitted to a rehydration protocol, being boiled in distilled water for 15 minutes, treated with 2% potassium hydroxide for two hours at room temperature and washed in tap water, being posteriorly dehydrated in an alcoholic series and stored in ethanol 70% (modified from Smith and Smith 1942).

**Table 1.** List of species investigated in micromorphological, anatomical, and histochemical studies. **T.** Trichomal elaiophores. **EU.** Epidermal unicellular elaiophores with vacuoles. **EO.** Epidermal overlapping elaiophores without vacuoles. **FA.** Fatty Acids. **Ph.** Phenolic compounds. **Ps.** Polysaccharides. **^1^** Species sampled in this study. **^2^** Species sampled by Arévalo-Rodrigues et al. (2020). **^3^** Species sampled by Lorenzo (1981). **^4^**Simpson (1989). **^5^**Aliscioni et al. (2019). **^6^** Miyashita et al. (1964). **^7^**Sanches et al. (2023). **^8^**Rao (1941). **^9^**Anderson (1980). ^10^Paiva et al. (2019). ^11^Stevens (2001, onwards). ^12^Amaral et al. (2017). ^13^Wurdack and Zartman (2019). ^14^Crockett (2010). ^15^Gama et al. (2016). ^16^Feng (2005).^17^Bonifácio et al. (2023).

Species	Voucher (Herbarium)	Clades	Anatomy	Histochemistry
*Caryocarbrasiliense* Cambess. (Caryocaraceae)^10^	Lombardi s.n. (BHCB53575)	–	EU	FA, Ph, Ps
Celastrales ^11^	–	–	–	–
*Clusiascrobiculata* Benoist (Clusiaceae)^12^	Ribeiro 1838 (INPA)	–	EU	FA, Ph, Ps
*Vantaneaspiritu-sancti* (Cuatrec.) K.Wurdack & Zartman (Humiriaceae)^13^	Silva et al. 1436 (US)	–	EU	Ph, Ps
*Hypericumperforatum* L. (Hypericaceae)^14^	–	–	EU	FA, Ph, Ps
*Phyllanthusurinaria* L. (Phyllanthaceae)^15^	T.S.S. Gama 6 (MFS)	–	–	–
Picrodendraceae ^11^	–	–	–	–
*Anchieteafrangulifolia* (Kunth) Melch. (Violaceae)^16^	Cuatrecasas 5477 (US)	–	EU	Ph
*Bergiaperennis* F.Muell. (Elatinaceae)^17^	Henshall 1479 (SP)	–	–	–
*Elatinegratioloides* A.Cunn. (Elatinaceae)^17^	Latz 7536 (SP)	–	–	–
*Byrsonimaincarnata* Sandwith^1^	Lima 05 (HUEFS)	Byrsonimoids	T, EU	FA, Ph, Ps
*Byrsonimaspicata* (Cav.) DC.^2^	Rodrigues 277 (SP)	Byrsonimoids	T, EU	absent
*Galphimiaaustralis* Chodat^1^	Almeida 767 (HUEFS)	Byrsonimoids	EU	absent
*Lophantheralactescens* Ducke^1^	Queiroz 5277 (HUEFS)	Byrsonimoids	EU	absent
*Pterandrapyroidea* A.Juss.^1^	Almeida 838 (JAR)	Byrsonimoids	EU	absent
*Verrucularinaglaucophylla* (A.Juss.) Rauschert^1^	Almeida 606 (HUEFS)	Byrsonimoids	EU	absent
*Burdachiaduckei* Steyerm.^1^	Giulietti 2591 (HUEFS)	Mcvaughioids	EU	absent
*Mcvaughiasergipana* Amorim & R.F.Almeida^1^	Amorim 8393 (HUEFS)	Mcvaughioids	EU	FA, Ph, Ps
*Barnebyaharleyi* W.R.Anderson^1^	Harley 54284 (HUEFS)	Barnebyoids	EU	FA, Ph, Ps
*Dinemandraericoides* A.Juss.^4^	Simpson 8310141 (TEX)	Ptilochaetoids	EU	absent
*Dinemagonumgayanum* A.Juss.^4^	Simpson 831082 (TEX)	Ptilochaetoids	EU	absent
*Ptilochaetabahiensis* Turcz.^1^	Almeida 858 (JAR)	Ptilochaetoids	EU	FA, Ph, Ps
*Bunchosiapernambucana* W.R.Anderson^1^	Mello 10765 (HUEFS)	Bunchosioids	EU	FA, Ph, Ps
*Thryallislatifolia* Mart.^1^	Almeida 687 (HUEFS)	Bunchosioids	EU	absent
*Tristellateiaaustralasiae* A.Rich.^8^	Rao s.n. (JCB)	Bunchosioids	EU	absent
*Hiraeahatschbachii* C.E.Anderson^1^	Almeida 548 (HUEFS)	Hiraeoids	EU	FA, Ph, Ps
*Lophopterysfloribunda* W.R.Anderson & C.C.Davis^7^	Sanches s.n. (UFV)	Hiraeoids	EU	FA, Ph, Ps
*Aliciaanisopetala* (A.Juss.) W.R.Anderson^1^	Almeida 890 (JAR)	Tetrapteroids	EO	absent
*Callaeumpsilophyllum* (A.Juss.) D.M.Johnson^1^	Almeida 724 (HUEFS)	Tetrapteroids	EO	absent
*Callaeumpsilophyllum* (A.Juss.) D.M.Johnson^1^	Almeida 734 (HUEFS)	Tetrapteroids	EO	absent
*Caroluschasei* (W.R.Anderson) W.R.Anderson^1^	Almeida 585 (HUEFS)	Tetrapteroids	EU	absent
*Christianellasurinamensis* (Koesterm.) W.R.Anderson^1^	Almeida 817 (HUEFS)	Tetrapteroids	EO	absent
*Dicellabracteosa* (A.Juss) Griseb.^1^	Cardoso 273 (HUEFS)	Tetrapteroids	EU	absent
*Glicophyllumcardiophyllum* (Nied.) R.F.Almeida^1^	Almeida 641 (HUEFS)	Tetrapteroids	EU	FA, Ph, Ps
*Heteropterysaenea* Griseb.^1^	Almeida 798 (HUEFS)	Tetrapteroids	EU	absent
*Niedenzuellalasiandra* (A.Juss.) R.F.Almeida^1^	Almeida 891 (RB)	Tetrapteroids	EU	absent
*Tetrapterysphlomoides* (Spreng.) Nied.^1^	Almeida 819 (HUEFS)	Tetrapteroids	EU	absent
*Tricomariausillo* Hook. & Arn.^5^	Aliscioni s.n. (CORD)	Tetrapteroids	EU	absent
*Amorimiarigida* (A.Juss.) W.R.Anderson^1^	Almeida 556 (HUEFS)	Malpighioids	EU	FA, Ph, Ps
*Aspidopterysconcava* (Wall.) A.Juss.^1^	Merrill 11601 (US)	Malpighioids	EU	absent
*Ectopopteryssoejartoi* W.R.Anderson^9^	Soejarto 3399 (US)	Malpighioids	EU	absent
*Malpighiaglabra* L.^6^	Miyashita 269–2 (HAW)	Malpighioids	EU	absent
*Mascagniasepium* (A.Juss.) Griseb.^1^	Almeida 822 (HUEFS)	Malpighioids	EU	absent
*Triaspismozambica* A.Juss.^1^	Robertson 6540 (US)	Malpighioids	EU	absent
*Aspicarpaharleyi* W.R.Anderson^2^	Hatschbach 67824 (HUEFS)	Stigmaphylloids	EO	absent
*Banisteriopsisadenopoda* (A.Juss.) B.Gates^2^	Almeida 813 (HUEFS)	Stigmaphylloids	EO	absent
*Banisteriopsisargyrophylla* (A.Juss.) B.Gates^2^	Almeida 808 (HUEFS)	Stigmaphylloids	EO	absent
*Banisteriopsislaevifolia* (A.Juss.) B.Gates^2^	Almeida 658 (HUEFS)	Stigmaphylloids	EO	absent
*Banisteriopsismalifolia* (Nees and Mart.) B.Gates^2^	Francener 1122 (SP)	Stigmaphylloids	EO	absent
*Banisteriopsismultifoliolata* (A.Juss.) B.Gates^2^	Demuner 3629 (SP)	Stigmaphylloids	EO	absent
*Banisteriopsisvariabilis* B.Gates^2^	Almeida 815 (HUEFS)	Stigmaphylloids	EO	absent
*Bronweniamegaptera* (B.Gates) W.R.Anderson & C.C.Davis^2^	Almeida 782 (HUEFS)	Stigmaphylloids	EU	absent
*Camareaaffinis* A.St.-Hil.^2^	Almeida 760 (HUEFS)	Stigmaphylloids	T	absent
*Camareahumifusa* W.R.Anderson^2^	Pastore 2310 (HUEFS)	Stigmaphylloids	T	absent
*Cottsiagracilis* (A.Gray) W.R.Anderson & C.C.Davis^2^	Sperry 597 (US)	Stigmaphylloids	T	absent
*Diplopteryslutea* (Griseb.) W.R.Anderson & C.C.Davis^2^	Almeida 210 (SP)	Stigmaphylloids	EO	absent
*Diplopteryspauciflora* (G.Mey.) Nied.^2^	de La Cruz 3134 (MG)	Stigmaphylloids	EU	absent
*Gallardoafischerii* Hicken^2^	Simon 891 (US)	Stigmaphylloids	EU	absent
*Gaudichaudiaalbida* Schltdl. & Cham.^2^	A.R. Molina 23061 (US)	Stigmaphylloids	EO	absent
*Gaudichaudiakrusei* W.R.Anderson^2^	s. col. (US2367483)	Stigmaphylloids	EO	absent
*Janusiaguaranitica* (A.St.-Hil.) A.Juss.^3^	Fulvio 164 (CORD)	Stigmaphylloids	EO	absent
*Peixotoahispidula* A.Juss.^2^	Almeida 818 (HUEFS)	Stigmaphylloids	EO	absent
*Sphedamnocarpusgalphimifolius* (A.Juss.) Szyszyl.^2^	Kimp 711 (US)	Stigmaphylloids	EO	absent
*Sphedamnocarpuspruriens* (A.Juss.) Szyszyl. ^2^	Strohback 53137 (US)	Stigmaphylloids	EU	absent
*Stigmaphyllonabutifolium* (A.Juss.) C.E.Anderson^2^	Hosaka 3378 (US)	Stigmaphylloids	EU	absent
*Stigmaphyllonblanchetii* C.E.Anderson^2^	Almeida 596 (HUEFS)	Stigmaphylloids	EU	FA, Ph, Ps
*Stigmaphyllongrandifolium* (A.Juss.) C.E.Anderson^2^	Kajwski 803 (US)	Stigmaphylloids	EU	absent
*Stigmaphyllonlalandianum* A.Juss.^2^	Almeida 816 (HUEFS)	Stigmaphylloids	EU	absent
*Stigmaphyllontimoriense* (DC.) C.E.Anderson^2^	Gray 303 (US)	Stigmaphylloids	EU	absent

### ﻿Light microscopy and histochemistry

Fixed or rehydrated samples were embedded using standard methods for Technovit historesin and sectioned at 2 µm thickness (Arévalo-Rodrigues et al. 2020). Sections were stained with toluidine blue/*p*-phenylenediamine (1% aqueous/1% isopropanol: methanol, 1:1) for identification of metachromasy/phenolic compounds and lipid identification, respectively (Feder and O’Brien 1968; Kivimäenpää et al. 2004), and subsequently mounted in water slides for structural analyses under light microscopy.

Regarding herbarium samples, it is possible to perform histochemical analysis of rehydrated samples, except for identifying low-weight lipophilic molecules, including phenolic-based molecules, essential oils, and alkaloids. Connective glands were histochemically characterised in ten genera, representing nine of the ten informal phylogenetic clades currently accepted for Malpighiaceae (Table 1). Additionally, not all rehydrated materials resulted in suitable samples for anatomical/histochemical analyses, aside from the reduced number of flowers in this study. To detect the main classes of compounds in the glands’ exudate, we used copper acetate/rubeanic acid for fatty acids (Ganter and Jolles 1969), PAS reaction for total polysaccharides (McManus 1948), and ruthenium red or tannic acid and ferric chloride for mucilage (Gregory and Baas 1989; Pizzolato 1977). The digital images were acquired with an Olympus BX53 compound microscope equipped with an Olympus I-Colour 5 digital camera and Image Pro Express 6.3 software.

### ﻿Scanning electron microscopy

Fixed anther samples from all 25 genera and 26 species of Malpighiaceae sampled (Table 1) were fully dehydrated using 100% ethanol, rinsed in a hexamethyldisilane (HMDS) series (33.3, 50.0, and 66.6% v/v in 100% ethanol), and then rinsed three times in 100% HMDS for 1 min each to dry the material (Jeger et al. 2009). Chemically dried and herbarium samples were mounted on stubs, sputter-coated with gold using a Leica ACE200 system, and viewed using a JEOL JSM 741F scanning electron microscope at 10 kV. All SEM-analysed specimens were imaged and compared with the micromorphology of the genera analysed for anatomy and histochemistry under light microscopy.

### ﻿Character coding and mapping

Character coding followed the recommendations of Sereno (2007) for morphological analyses. Primary homology hypotheses (i.e., Ad doc hypothesis; De Pinna 1991; Ochoterena et al. 2019) were proposed for a total of 46 genera and 59 species of Malpighiaceae: 25 genera and 26 species sampled in this study, and 21 genera and 33 species of Malpighiaceae and nine families of Malpighiales sampled from the specialised literature (Tables 1, 2). Three micromorphological characters were scored, coded, and optimised (i.e. ancestral state reconstruction analysis) using the Maximum Likelihood criterium implemented on Mesquite 2.73 (Maddison and Maddison 2009) on a trimmed consensus tree from the molecular phylogeny of Malpighiaceae published by Davis and Anderson (2010; TreeBase accession 10998), evidencing a single tip per genus and the ten informal clades proposed by de Almeida and van den Berg (2021).

**Table 2.** Morphological matrix, including two morphoanatomical characters and a single histochemical character, scored and coded based on our results and the specialised literature. **Character 1.** Stamen, connective, elaiophore, type: (0) Trichomal, (1) Epidermal unicellular elaiophores with vacuoles, (2) Epidermal overlapping elaiophores without vacuoles, (?) missing data. **Character 2.** Stamen, gland, position: (0) filaments, (1) connectives, (2) absent. **Character 3.** Stamen, gland, exudate, type: (0) oil, (1) resin, (2) nectar, (-) not applicable, (?) missing data. Taxa highlighted in bold represent phylogenetic outgroups.

Genera/Families	Character 1	Character 2	Character 3
**Caryocaraceae**	–	0	0
**Celastrales**	–	2	–
**Clusiaceae**	–	1	1
**Humiriaceae**	–	1	2
**Hypericaceae**	–	1	1
**Phyllanthaceae**	–	2	–
**Picrodendraceae**	–	2	–
**Violaceae**	–	1	2
***Bergia* L. (Elatinaceae)**	–	2	–
***Elatine* L. (Elatinaceae)**	–	2	–
*Acmanthera* (A.Juss.) Griseb.	?	?	?
*Acridocarpus* Guill., Perr. & A.Rich.	?	?	?
*Adelphia* W.R.Anderson	?	?	?
*Alicia* W.R.Anderson	2	1	0
*Amorimia* W.R.Anderson	1	1	0
*Aspicarpa* Rich.	2	1	0
*Aspidopterys* A.Juss. ex Endl.	1	1	0
*Banisteriopsis* C.R.Rob.	2	1	0
*Barnebya* W.R.Anderson & B.Gates	1	1	0
*Blepharandra* Griseb.	?	?	?
*Brachylophon* Oliv.	?	?	?
*Bronwenia* W.R.Anderson & C.C.Davis	1	1	0
*Bunchosia* Rich. ex Kunth	1	1	0
*Burdachia* A.Juss.	1	1	0
*Byrsonima* Rich. ex Kunth	0/1	1	0
*Calcicola* W.R.Anderson	?	?	?
*Callaeum* Small	2	1	0
*Camarea* A.St.-Hil.	0	1	0
*Carolus* W.R.Anderson	1	1	0
*Caucanthus* Forssk.	?	?	?
*Christianella* W.R.Anderson	2	1	0
*Coleostachys* A.Juss.	?	?	?
*Cordobia* Nied.	?	?	?
*Cottsia* Dubard & Dop	0	1	0
*Diacidia* Griseb.	?	?	?
*Diaspis* Nied.	?	?	?
*Dicella* Griseb.	1	1	0
*Digoniopterys* Arènes	?	?	?
*Dinemagonum* A.Juss.	1	1	0
*Dinemandra* A.Juss.	1	1	0
*Diplopterys* A.Juss.	1/2	1	0
*Echinopterys* A.Juss.	?	?	?
*Ectopopterys* W.R.Anderson	1	1	0
*Excentradenia* W.R.Anderson	?	?	?
*Flabellaria* Cav.	?	?	?
*Flabellariopsis* R.Wilczek	?	?	?
*Gallardoa* Hicken	1	1	0
*Galphimia* Cav.	1	1	0
*Gaudichaudia* Kunth	2	1	0
*Glandonia* Griseb.	1	1	0
*Glicophyllum* R.F.Almeida	1	1	0
*Heladena* A.Juss.	?	?	?
*Henleophytum* H.Karst.	?	?	?
*Heteropterys* Kunth	1	1	0
*Hiptage* Gartn.	?	?	?
*Hiraea* Jacq.	1	1	0
*Janusia* A.Juss.	2	1	0
*Jubelina* A.Juss.	?	?	?
*Lasiocarpus* Liebm.	?	?	?
*Lophanthera* A.Juss.	1	1	0
*Lophopterys* A.Juss.	1	1	0
*Madagasikaria* C.C.Davis	?	?	?
*Malpighia* L.	1	1	0
*Malpighiodes* Nied.	?	?	?
*Mascagnia* (Bertero ex DC.) Bertero	1	1	0
*Mcvaughia* W.R.Anderson	1	1	0
*Mezia* Schwacke ex Nied.	?	?	?
*Microsteira* Baker	?	?	?
*Mionandra* Griseb.	?	?	?
*Niedenzuella* W.R.Anderson	1	1	0
*Peixotoa* A.Juss.	2	1	0
*Psychopterys* W.R.Anderson & S.Corso	?	?	?
*Pterandra* A.Juss.	1	1	0
*Ptilochaeta* Turcz.	1	1	0
*Rhynchophora* Arènes	?	?	?
*Spachea* A.Juss.	?	?	?
*Sphedamnocarpus* Planch. ex Benth. & Hook. f.	1/2	1	0
*Stigmaphyllon* A.Juss.	1	1	0
*Tetrapterys* Cav.	1	1	0
*Thryallis* Mart.	1	1	0
*Triaspis* Burch.	1	1	0
*Tricomaria* Gillies ex Hook. & Arn.	1	1	0
*Tristellateia* Thouars	1	1	0
*Verrucularina* Rauschert	1	1	0

## ﻿Results

**Figure 2.**
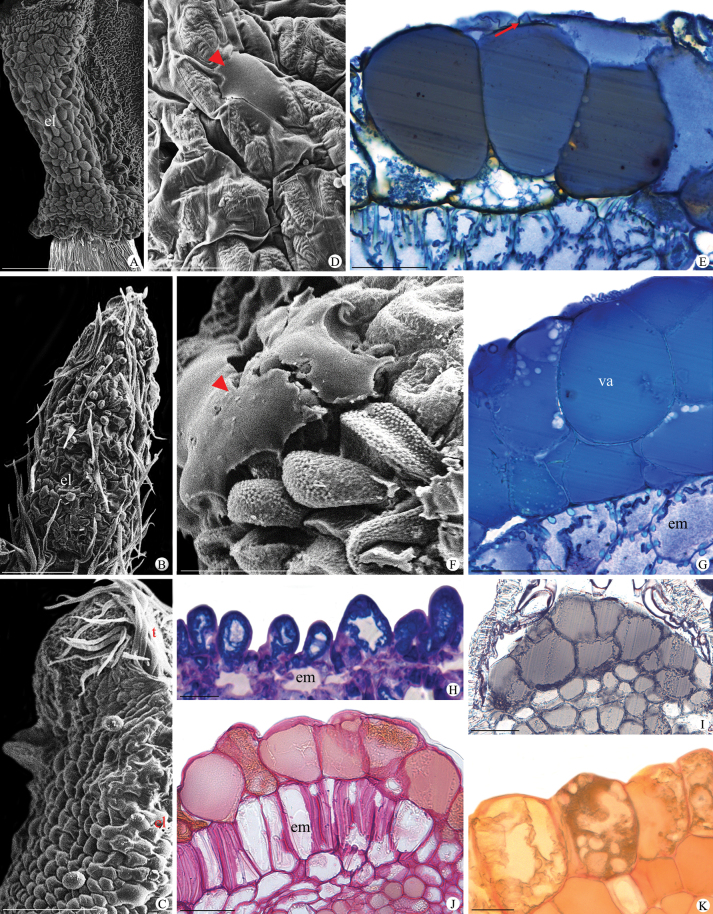
Staminal elaiophores of Malpighiaceae species **A** smooth globose cells comprise all the connectives of *Lophantheralactescens***B** in *Niedenzuellalasiandra*, non-secretory trichomes occur in all the anther epidermis, while in **C***Caroluschasei*, they permeate only the anther’s edge **D***Pterandrapyroidea*. Detail of the exudate of the globose epidermal cells (arrowhead in D, F) **E** the unicellular globose epidermal cells have a dense vacuole in *Amorimiarigida*; note the cuticle detachment (arrow). (**F–I**) *Callaeumpsilophyllum*. The latter species have elaiophores formed by overlapping globose epidermal cells with a dense vacuole in the connective **F, G** and unicellular trichomal elaiophores in the anther epidermis **H** The unicellular trichomal elaiophores exhibit lipid droplets in the protoplast **H**. **J, K***Bunchosiapernambucana***I–K** fatty acids, polysaccharides and phenolic compounds constitute the secretion inside the cell. el = elaiophores; em = endothecium; t = trichomes; va=vacuole. SEM (**A–D, F**). TBO+*p*-phe (**E, G–H**). AA (**J**). PAS (**J**). VR (**K**). Scale bars: 250 µm (**B**); 200 µm (**A, C**); 100 µm (**D, F**); 50 µm (**E, G–K**).

The connectives of all species analysed under light microscopy and SEM showed elaiophores within their lining tissue (Fig. 2A–G, I–K, Table 1). We classified these connectives into three distinct morphotypes: 1. trichomal, composed of secretory papillae in some species of *Byrsonima*, *Camarea*, and *Cottsia* (Fig. 2H, Table 1); 2. epidermal, comprising unicellular globose cells with vacuoles showing polyphenols with different aspects (i.e., granulose, dense, or both), and also the occurrence of many lipid droplets inside them in 33 genera of Malpighiaceae (Fig. 2A–E, Table 1); and 3. epidermal, comprising overlapping globose epidermal cells mainly found in ten genera of Malpighiaceae (Fig. 2F–G, Table 1). The histochemical analyses identified the occurrence of fatty acids (Fig. 2I, Table 1), polysaccharides (Fig. 2J, Table 1), and phenolic compounds (Fig. 2K, Table 1) inside the glandular tissue from the connectives of the analysed genera, and presumably, the secretion of these staminal glands is heterogeneous.

**Figure 3.**
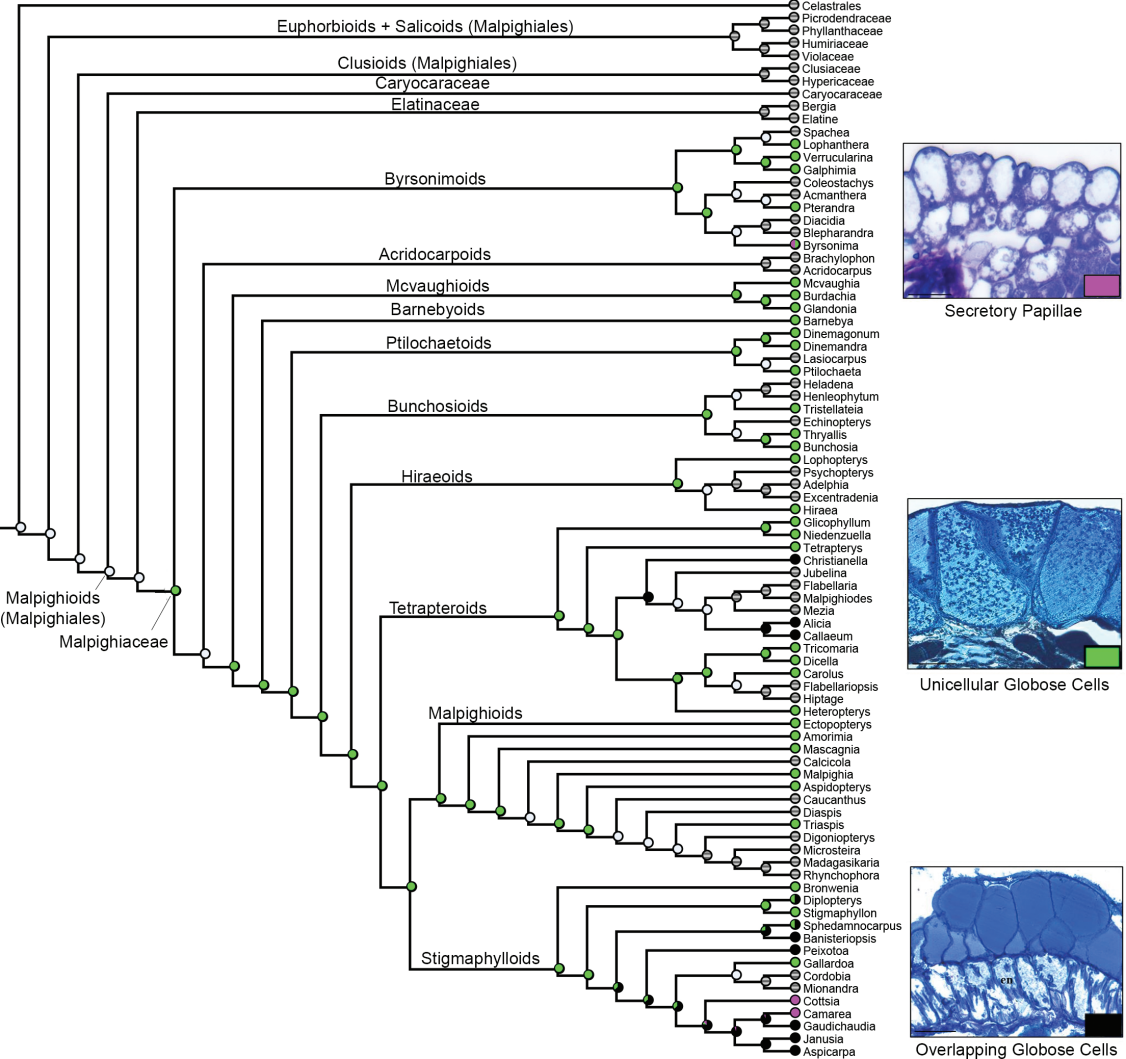
Character-mapping analysis showing the evolution of the three identified types of connective elaiophores by this study in Malpighiaceae. Gray dots represent not applicable (missing data) character states.

**Figure 4.**
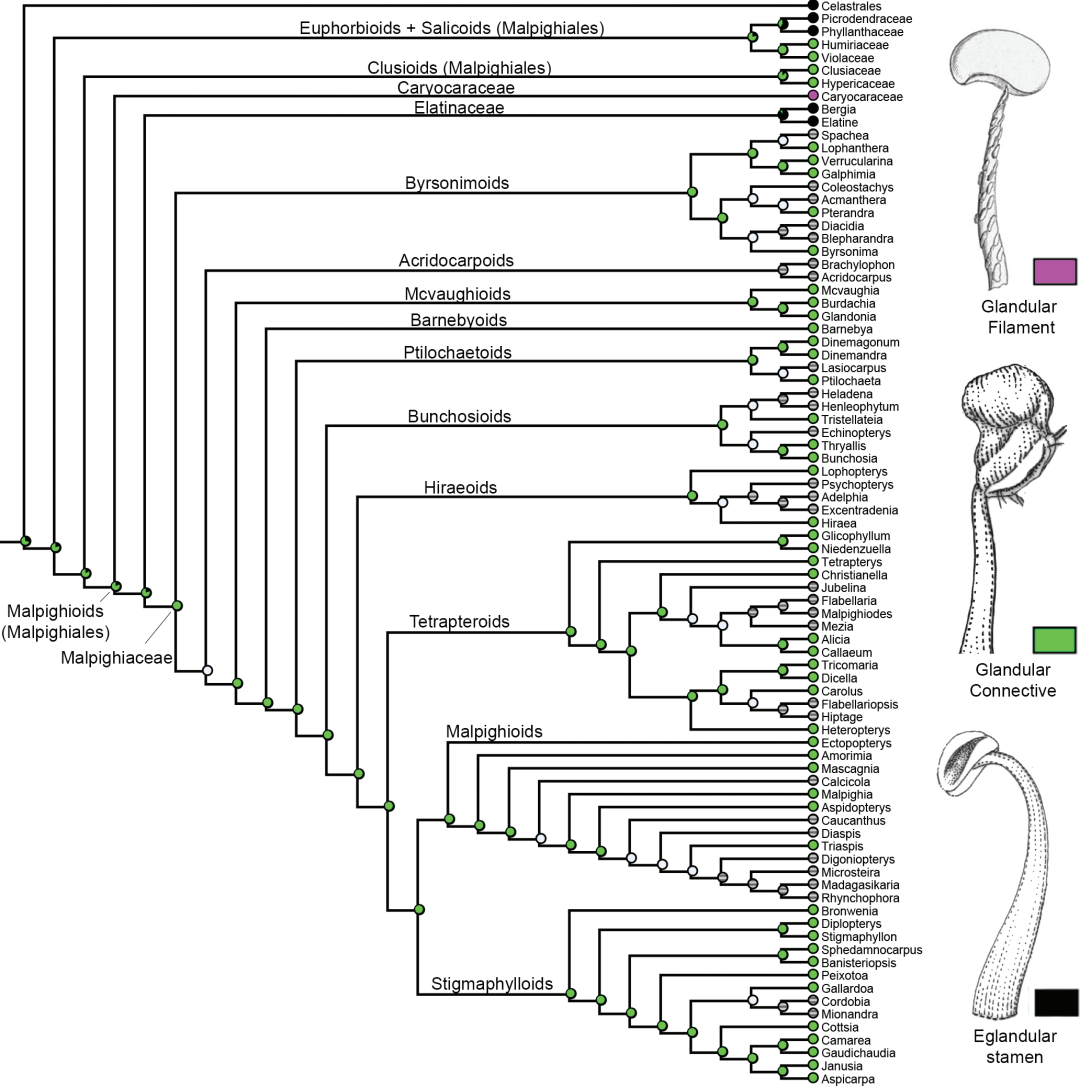
Character-mapping analysis showing the evolution of the three identified types of stamen glands and their respective place of insertion (i.e., on filaments, on connectives or entirely eglandular) by this study in Malpighiaceae. Gray dots represent not applicable (missing data) character states.

**Figure 5.**
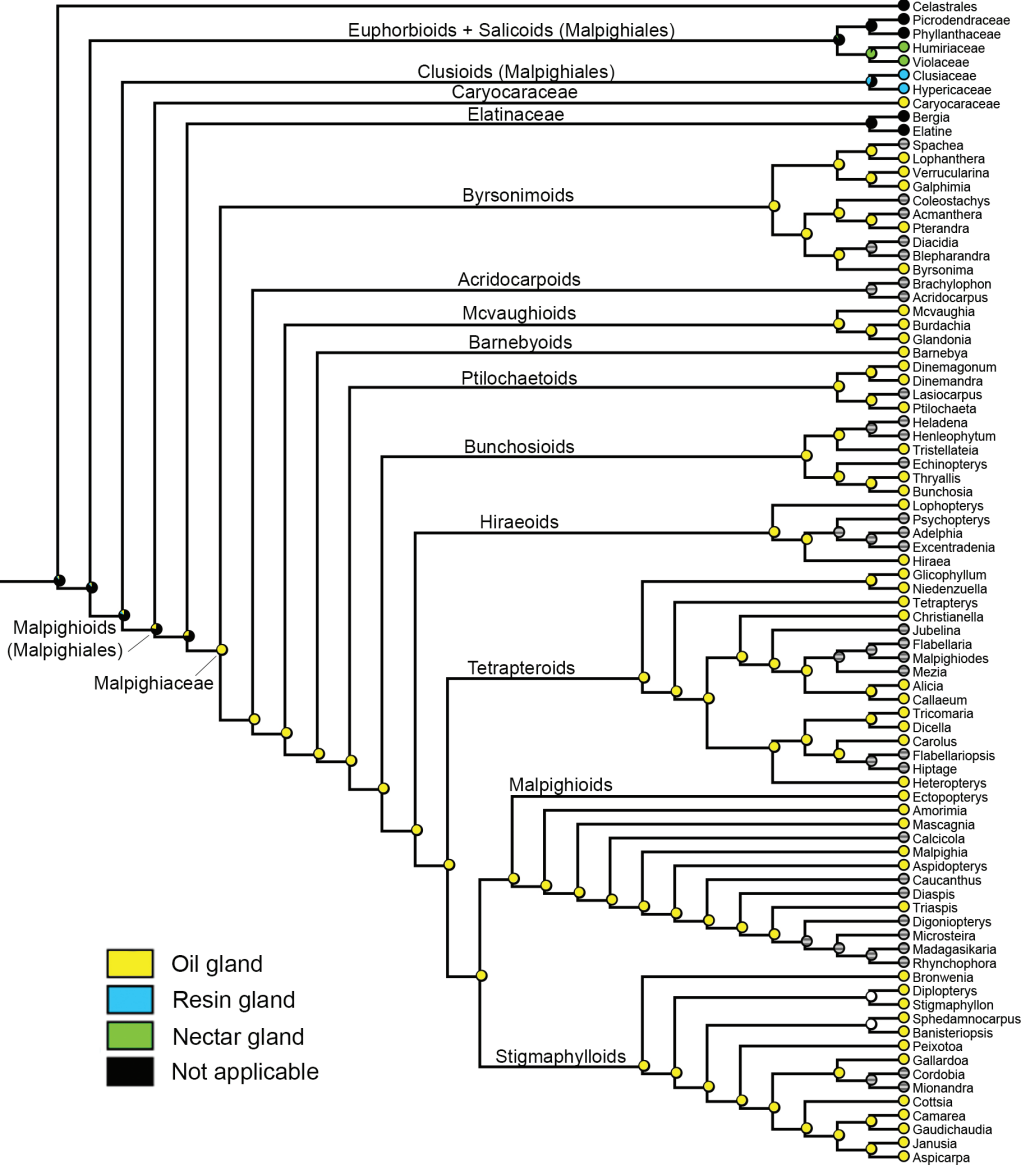
Character-mapping analysis showing the evolution of the four identified types of stamen glands exudate (i.e., oil gland, resin gland, nectar gland or eglandular) in Malpighiales. Grey dots represent missing data, and black dots represent not applicable character state (i.e., taxa with eglandular stamens, which do not produce any exudate, being coded as not applicable).

Three micromorphological characters were scored and coded for our sampling (see Table 2). Unicellular globose cells were recovered as the ancestral state in Malpighiaceae connectives, being retained in most lineages of this family (Fig. 3). Secretory papillae were recovered as a homoplasy in the connectives of *Byrsonima*, *Cottsia* and *Camarea* (Fig. 3). Overlapping globose cells were also recovered as a homoplasy for the most recent common ancestor of the *Christianella* and *Banisteriopsis* clades, within the Tetrapteroids and Stigmaphylloids, respectively (Fig. 3). Glandular connectives were recovered by us as the ancestral state for Malpighiales (Fig. 4). Glandular filaments were recovered as a synapomorphy for Caryocaraceae (Fig. 4). Eglandular stamens were recovered as homoplastic synapomorphies for Elatinaceae, the Euphorbioids (represented in our analysis by Picrodendraceae + Phyllanthaceae), and Celastrales (Fig. 4). Regarding the nature of the staminal gland exudate, glandular connectives producing non-volatile oils were recovered as a synapomorphy for Malpighiaceae (Fig. 5), while glandular filaments producing non-volatile oils were recovered as a synapomorphy for Caryocaraceae (Fig. 5). Glandular connectives producing resin were recovered as a probable synapomorphy of the Clusioids (Clusiaceae + Hypericaceae; Fig. 5). Finally, glandular connectives producing nectar were recovered as a probable synapomorphy of the Salicoids (Humiriaceae + Violaceae).

## ﻿Discussion

### ﻿Evolution of connective elaiophores in Malpighiaceae

**Figure 6.**
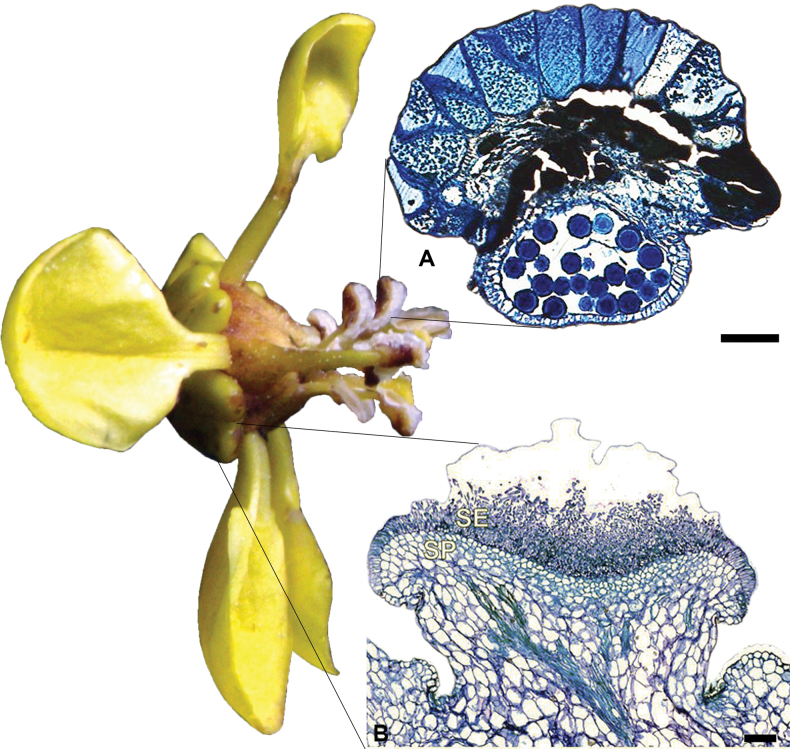
Comparison between staminal and sepal elaiophores of a Malpighiaceae flower **A** transversion section of an anther of *Stigmaphyllonblanchetii* C.E.Anderson **B** longitudinal section of sepal elaiophore of *Mcvaughiasergipana* Amorim & R.F.Almeida. Flower of *Heteropterysoberdanii* Amorim is shown in a side view, evidencing the location of elaiophores in different organs (**A** modified from Arévalo-Rodrigues et al. 2020; **B** modified from Almeida et al. 2019; photograph of *H.oberdanii* by R.F.Almeida).

The glandular connectives observed in this study in all analysed species of Malpighiaceae were characterised as elaiophores, occurring as epidermal cells or papillae (i.e., trichomal elaiophores). The connective elaiophores described in this study were mainly formed by globose cells, with distinct anatomical features from those found in the sepals, as reported by Arévalo-Rodrigues et al. (2020; Fig. 6). Sepal elaiophores are composed of a palisade epidermis bearing a thick cuticle with vascularised parenchyma (Vogel 1974; Castro et al. 2001; Possobom et al. 2015; Araújo and Meira 2016; Possobom and Machado 2017; Aliscioni et al. 2022). Alternatively, connective elaiophores of Malpighiaceae comprise only one or two layers of globose epidermal cells or papillae (Possobom et al. 2015; Arévalo-Rodrigues et al. 2020; Avalos et al. 2020; Fig. 6). Nonetheless, trichomal elaiophores (i.e., papillae) in the connectives of Malpighiaceae have only recently been reported (Arévalo-Rodrigues et al. 2020). Despite epidermal and trichomal elaiophores sometimes co-occurring in the same plant organ (Pansarin et al. 2009), their occurrence in the same family is uncommon in flowering plants, being only reported for Malpighiaceae, Iridaceae, Orchidaceae, Plantaginaceae and Scrophulariaceae so far (Vogel 1974, 1984; Vogel and Machado 1991; Cosacov et al. 2009; Renner and Schaefer 2010).

The heterogeneous connective elaiophore secretion produced in Malpighiaceae comprises a mixture of lipids, polysaccharides and phenolic compounds. The same exudate composition was found in the glandular connectives of *Diplopteryspubipetala*, *Stigmaphyllonbonariense* (Hook. & Arn.) C.E.Anderson, and *S.jatrophifolium* A.Juss. (Possobom et al. 2015; Avalos et al. 2020). A heterogeneous secretion mainly comprising non-volatile oils and polysaccharides is also produced by epidermal cells in the sepal elaiophores of Malpighiaceae (Vogel 1974; Lobreau-Callen 1989; Vinson et al. 1997; Castro et al. 2001; Possobom and Machado 2017; Aliscioni et al. 2022). This heterogeneous composition grants the exudate better fluidity, facilitating its collection by pollinators (Pansarin et al. 2009) being also observed in the nectar and stigmatic secretions of other flowering plant families, including Malpighiaceae (Endress 1994; Fahn 2000; Aliscioni et al. 2018). This fluid exudate of connective glands in Malpighiaceae resembles the fluid resin secreted by the connective glands of Calophyllaceae and the filaments of Clusiaceae and future in depth histochemistry studies should shed some light into their homology (Amaral et al. 2017; Cabral et al. 2021). Additionally, phenolic compounds show a central role as antioxidants and are secondarily astringent and toxic (Bruneton 1999; Simões et al. 2004), protecting floral resources from pathogens. *Monoeca* bees have been reported to collect pollen with the anthers’ exudate (Possobom et al. 2015). The connective elaiophores have the primary function of adhering pollen grains to the pollinator’s body, but they might also show a secondary chemical attraction function for these oil-collecting bees (Possobom and Machado 2017; Avalos et al. 2020).

The character-mapping analysis recovered unicellular globose cells (i.e., epidermal elaiophores) producing mainly non-volatile oil in the connectives of Malpighiaceae as a new synapomorphy for this family. This result was only possible due to our comprehensive analysis sampling 46 genera (out of 75) from nine of the ten informal phylogenetic clades currently recognised in Malpighiaceae (see Almeida and van den Berg 2021; Almeida et al. 2023). In fact, unicellular 2-branched hairs, conspicuous sepal elaiophores and clawed petals are already recognised as morphological synapomorphies for Malpighiaceae (APG IV 2016; Stevens 2001, onwards), with connective elaiophores proposed here as a fourth morphological synapomorphy for this family. Nonetheless, additional anatomic, histochemical and SEM studies sampling all the remaining genera of Malpighiaceae (see Table 2 for additional information) are still needed to better explore the evolutionary patterns of connective elaiophores structure and exudate composition in this family.

### ﻿Evolution of staminal glands in Malpighiales

Based on our sampling, the character-mapping analysis recovered glandular connectives as a possible synapomorphy for Malpighiales, one of Rosids’ major extant orders, with 42 families and ~16,000 species with a mostly pantropical distribution, exhibiting remarkable morphological and ecological diversity (Xi et al. 2012). However, further studies focusing on characterising glandular tissue in the stamens of most families comprising Malpighiales are still needed. In fact, we were only able to sample nine out of the 42 families currently accepted in this order (see Table 1). Although strongly monophyletic, relationships between Malpighiales families remain elusive, and no consensus has been reached even in the phylogenomic era (Davis et al. 2005; Wurdack and Davis 2009; Xi et al. 2012; Cai et al. 2019). Even though unambiguous morphological synapomorphies have not been proposed yet for Malpighiales, the order is generally characterised by a combination of homoplastic characters (i.e., paracytic stomata, leaf-blades with toothed margins, diverse extrafloral glands, ovules with thin, slender nucelli and endothelium, and dry stigmas; Stevens 2001, onwards), possibly including staminal glands.

The character-mapping analysis also recovered different types of staminal gland exudate as possible synapomorphies for some major clades of Malpighiales. This order currently comprises four major clades (i.e., Clusioids, Euphorbioids, Malpighioids, and Salicoids) recognised by different studies (Xi et al. 2012; Cai et al. 2019). However, no synapomorphies have ever been proposed for these four major clades of Malpighiales (Xi et al. 2012; Stevens 2001, onwards). The most recent common ancestor for the Clusioids had resin connective glands recovered in our analysis as a possible synapomorphy for this clade. Several types of exudates are secreted by the connective glands of Clusioids, such as wax and resins in Calophyllaceae (Crockett 2010), Clusiaceae (Crockett 2010; Amaral et al. 2017), and Hypericaceae (Crockett 2010). Currently, the Clusioids only show basifixed anthers as a homoplastic morphological character uniting its members (Silva-Batista et al. 2021).

The Euphorbioids had eglandular stamens recovered as a possible homoplasy shared with Elatinaceae and Podostemaceae in our analysis probably representing a reversal due to the specialised aquatic life form. The Euphorbioids are currently circumscribed by homoplastic characters such as plants often monoecious, flowers small, often imperfect, and 3-merous or not, ovules 1–2/carpel, inner integument usually thicker than outer, epitropous, fruit a part-septicidal + loculicidal capsule/schizocarp, cotyledons longer and broader than radicle (Stevens 2001, onwards). The Euphorbioids comprise five additional families (i.e., Euphorbiaceae, Ixonanthaceae, Linaceae, Peraceae, and Rafflesiaceae) that had never had their stamens anatomically studied, focusing on glandular tissues. In fact, most of these families are pantropically distributed or are difficult to analyse due to their rarity (i.e., Rafflesiaceae; POWO 2023; Stevens 2001, onwards). Further studies are urgently needed in these Euphorbioid families to corroborate the absence of staminal glands in this major clade of Malpighiales.

In our analysis, the Malpighioids (Malpighiales) had oil glandular stamens recovered as a possible synapomorphy. This major clade of Malpighiales had only three (out of 11) of its families anatomically explored regarding staminal glands. Malpighiaceae has already been the subject of staminal glands anatomical studies by several authors (Possobom et al. 2015; Arévalo-Rodrigues et al. 2020; Avalos et al. 2020; this study). On the other hand, Elatinaceae comprises only two genera, *Bergia* and *Elatine*, which were recently sampled in a comprehensive anatomical study by Bonifácio et al. (2023) and seemed to present, indeed, eglandular stamens in most of its species, except for *Elatinelindbergii* Rohrb. These authors performed histochemical tests for *E.lindbergii* indicating the presence of phenolic compounds, but they failed to test the exudate of these glands for lipids. This family is also an aquatic family, just like Podostemaceae, even though not that morphologically specialised and having cleistogamous (sometimes flowering underwater) and/or apomictic flowers. Caryocaraceae have also been anatomically studied by several authors regarding their staminal glands (Dickison 1990; Matthews and Endress 2011; Sousa-Paiva et al. 2019). In fact, the Malpighioids (Malpighiales) show few circumscribing homoplasies, such as gynoecium with longitudinal bulges above the placentae, outer integument 3–7 cells across, and inner integument 5–10 cells across (Stevens 2001, onwards). Nonetheless, further studies are still needed on the eight remaining families of the Malpighioids to test the relevance of staminal oil glands as possible synapomorphies or homoplasies circumscribing this major clade of Malpighiales.

Finally, the Salicoids had nectar connective glands recovered as a synapomorphy in our analysis. Nectar connective glands have recently been suggested as a new morphological synapomorphy for Humiriaceae (Wurdack and Zartman 2019). However, this exudate is also recorded in the filament or connective glands of Violaceae within the Salicoids (Feng 2005; Wurdack and Zartman 2019). In fact, only persistent endosperm has ever been proposed as a morphological homoplasy to circumscribe the Salicoids, making the nectar connective glands reported in this study a prominent character to further explore in this group (Stevens 2001, onwards). Classical morphological studies had long suggested that there was a group that included Salicaceae, Achariaceae, Violaceae, Flacourtiaceae, and Passifloraceae and its segregates, Malesherbiaceae and Turneraceae, in part because of their common possession of parietal placentation, some sort of corona or scales in the flower, nectaries outside the stamens, etc. (e.g. Cronquist 1981). However, additional studies in members of Achariaceae, Goupiaceae, Passifloraceae s.lat., and Salicaceae s.lat. are still needed to better explore the presence, structure, and nature of connective glands in these Salicoid families.

## ﻿Conclusions

Connective elaiophores are proposed, for the first time, as a new synapomorphy for Malpighiaceae based on the characterisation and evolution of 46 genera of this family. Different types of connective glands (i.e., epidermal or trichomal elaiophores) were recovered as homoplasies for the *Christianella* and *Banisteriopsis* clades (i.e., overlapping globose epidermal elaiophores) and the genera *Byrsonima*, *Camarea* and *Cottsia* (i.e., trichomal elaiophores). Their position in the stamens (i.e., connectives or filaments) and exudate type were useful in evidencing evolutionary patterns within the Malpighiales sampling used in this study. Nonetheless, connective and filament glands in Malpighiales are yet to be evolutionarily studied in a broad context or even synoptically surveyed since only nine families (from 36) have any anatomical information available in the literature. Our results only represent the first glance at the potential of these staminal glands in aiding the systematics of Malpighiales and its major clades.

## ﻿Additional information

### Author ORCIDs

Rafael Felipe de Almeida https://orcid.org/0000-0002-9562-9287

Gustavo Arévalo-Rodrigues https://orcid.org/0000-0002-1860-1707

Isa L. de Morais https://orcid.org/0000-0001-8748-9723

Poliana Cardoso-Gustavson https://orcid.org/0000-0003-0978-9389

### Data availability

All of the data that support the findings of this study are available in the main text.
